# Synthesis and evaluation of the effects of solid lipid nanoparticles of ivermectin and ivermectin on cuprizone-induced demyelination via targeting the TRPA1/NF-kB/GFAP signaling pathway

**DOI:** 10.22038/IJBMS.2023.71309.15493

**Published:** 2023

**Authors:** Tayebeh Noori, Ahmad Reza Dehpour, Seyede Darya Alavi, Seyede Zahra Hosseini, Sina Korani, Antoni Sureda, Jamileh Esmaeili, Samira Shirooie

**Affiliations:** 1 Pharmaceutical Sciences Research Center, Health Institute, Kermanshah University of Medical Sciences, Kermanshah, Iran; 2 Department of Pharmacology, School of Medicine, Tehran University of Medical Sciences, Tehran, Iran; 3 Experimental medicine research center, Tehran University of medical sciences, Tehran, Iran; 4 Student Research Committee, Faculty of Pharmacy, Kermanshah University of Medical Sciences, Kermanshah, Iran; 5 Research Group on Community Nutrition and Oxidative Stress (NUCOX) and Health Research Institute of Balearic Islands (IdISBa), University of Balearic Islands-IUNICS, Palma de Mallorca E-07122, Balearic Islands, Spain; 6 CIBER Fisiopatología de la Obesidad y Nutrición (CIBEROBN), Instituto de Salud Carlos III (ISCIII), 28029 Madrid, Spain

**Keywords:** Cuprizone, Demyelinating diseases, Inflammation, Ivermectin, Multiple sclerosis, Nano-Ivermectin, solid lipid nanoparticle, TRPA1 cation channel

## Abstract

**Objective(s):**

Multiple sclerosis (MS) is a chronic disease of the central nervous system (CNS) and its cause is unknown. Several environmental and genetic factors may have roles in the pathogenesis of MS. The synthesis of solid lipid nanoparticles (SLNs) for ivermectin (IVM) loading was performed to increase its efficiency and bioavailability and evaluate its ability in improving the behavioral and histopathological changes induced by cuprizone (CPZ) in the male C57BL/6 mice.

**Materials and Methods:**

Four groups of 7 adult C57BL/6 mice including control (normal diet), CPZ, IVM, and nano-IVM groups were chosen. After synthesis of nano-ivermectin, demyelination was induced by adding 0.2% CPZ to animal feed for 6 weeks. IVM and nano-IVM (1 mg/kg/day, IP) were given for the final 14 days of the study. At last, behavioral tests, histochemical assays, and immunohistochemistry of TRPA1, NF-kB p65, and GFAP were done.

**Results:**

The time of immobility of mice in the IVM and nano-IVM groups was reduced compared to the CPZ group. Histopathological examination revealed demyelination in the CPZ group, which was ameliorated by IVM and nano-IVM administration. In IVM and nano-IVM groups corpus callosum levels of TRPA1, NF-kB p65, and GFAP were decreased compared to the CPZ group. In the IVM and nano-IVM groups, the levels of MBP were significantly higher than in the CPZ group.

**Conclusion:**

The results evidenced that IVM and nano-IVM administration is capable of reducing demyelination in mice.

## Introduction

Multiple sclerosis (MS) is a chronic inflammatory and autoimmune disease of the central nervous system (CNS) (1, 2). Macrophages, B and T cells, and their autoreactive response to the myelin sheath near the axons of the CNS are involved in its pathogenesis (3, 4). Inflammation and oxidative stress are crucial factors in the pathogenesis of MS (5). Oxidative stress, caused by an imbalance between pro-oxidant and antioxidant mechanisms, is one of the most common features in the brain of MS patients (5, 6) along with raised levels of reactive oxygen species (ROS) in cerebrospinal fluid (CSF) of these patients (7). To study MS in animal models, the most common method is to trigger the pathology cuprizone (CPZ) (8). CPZ is a copper chelator whose use induces programmed death in oligodendrocyte cells, chronic inflammation, increased astrocyte and microglial cell activity, and ultimately, myelin destruction (9). Trapping or decreasing copper levels due to chelation leads to increased intracellular copper, which is a possible mechanism for CPZ toxicity (10). 

Transient receptor potential channels (TRP channels) play an essential role in physiological processes such as chemical sensing, and pain, and also mediates the release of cytokines (11). These channels regulate inflammation through sensory function and the release of neuropeptides. TRP channel subfamilies V, M, and A members such as TRPV1, TRPV4, TRPM3, TRPM8, and TRPA1 are subsets of TRP channels involved in immune responses and inflammation (11). TRPA1 is a non-selective cation channel with a high permeability to Ca^2+^ and is involved in the inactivation of chemically activated receptors. TRPA1 is a sensor for harmful stimuli and temperatures (12, 13), which is expressed in a variety of tissues, including the rodent cortex, dorsal root ganglia, caudal nucleus, colon innervations, bladder, and rat pancreatic beta cells (14-16). In the CNS, the expressions of TRPA1 in the cortex, caudate trigeminal nucleus, and hippocampal astrocytes have been reported in several studies (17, 18). Furthermore, the expression of TRPA1 channels in mature astrocytes has been shown to play an important role in resting intracellular Ca^2+^ levels (18). The presence of TRPA1 in rodent hippocampal astrocytes is effective in inhibiting synapses and regulating basal calcium levels (18, 19). It has been indicated that knockout of the TRPA1 gene leads to a reduction in adult oligodendrocyte apoptosis in CPZ-induced demyelination mice (19, 20), thus it can be concluded that astrocytic TRPA1 has a role in the regulation of apoptosis via Bak expression, pathways of mitogen-activated protein kinase (MAPK), and transcription factor c-Jun (21, 22). Inhibition of TRPA1 has been shown to protect neuroglial cells from demyelination, suggesting that TRPA1 could be a suitable target for treating MS (16). Though scientific advances have led to a better understanding of the pathogenesis and pathophysiology of MS, no definitive cure for the underlying cause has been found. The treatment goal of “first-line therapies” is to prevent the onset or recurrence of the disease. The most common treatment is the administration of anti-inflammatory drugs that suppress the immune system and, subsequently, inactivate or slow down the activation of all subtypes of T cells (23). 

Ivermectin (IVM), an antiparasitic drug, is used in the treatment of cutaneous larva migrans, scabies, filariasis, onchocerciasis, ascariasis, and strongyloidiasis (24). The results of clinical trials have shown that topical IVM reduces the number of inflammatory sores by having anti-inflammatory effects (25, 26). IVM is a highly hydrophobic drug (27), with low water solubility and its bioavailability is very low, particularly in ruminants (28). The oral bioavailability of lipophilic compounds has been shown to increase through lipid nanoparticles such as solid lipid nanoparticles (SLNs) (29). SLNs and nanostructured lipid carriers, due to their natural components, have been introduced as potentially attractive choices (30). SLNs have an average size of 50 to 1000 nm and spherical morphology and are composed of solid-phase lipids and surfactants (31). Lipid components of SLNs are solid at both body and ambient temperatures, and they can be pure triglycerides, glyceride mixtures, or even waxes. Proper selection of lipids and surfactants affects their physicochemical properties and quality, such as particle size, loading rate, and drug release pattern (32). High drug loading, increased drug stability, biotoxicity, carrying of hydrophobic or hydrophilic drugs, or better diagnostics are some of the benefits of nanocarriers. SLNs are made from solid lipids and stabilized through surfactants (33, 34). Altogether, the present study aimed to design a drug delivery system based on SLNs for the delivery of IVM to treat MS via targeting the TRAP1/NF-kB/GFAP signaling pathway.

## Materials and Methods

***Materials***

IVM, stearic acid (SA), dioctyl sulfosuccinate sodium salt (AOT), and glycerol tripalmitate (TPG) were purchased from Sigma Aldrich and, CPZ was obtained from Merck KGaA, Germany. The following antibodies were used for immunohistochemistry assay: mouse monoclonal TRPA1 antibody (ANKTM1 (C-5): SANTA CRUZ -376495), NF-kappaB p65 ((F-6): SANTA CRUZ -8008), GFAP monoclonal antibody (E-AB-22022 from Elabscience), anti-myelin basic protein antibody (ab40390 from Abcam), and m-IgGκ BP-HRP (SANTA CRUZ-516102) as the secondary antibody. 

***Preparation of solid lipid nanoparticles (SLNs)***

IVM-loaded SLNs were designed through new combined ultrasonic solvent evaporation procedures (35). Briefly, 4 mg of glycerol trimethoprim, 5 mg of stearic acid (lipid), 4 mg IVM, and 4 mg AOT (as surfactant), were dissolved in ethanol (1 ml). Using a probe sonicator (Sonopuls Ultrasonic Homogenizer HD 2070, Berlin), the lipid phase was dispersed in 40 ml aqueous phase (for 4 min). Then, the organic solvent was evaporated using stirring (900 rpm, 5 min) to obtain solid lipid nanoparticles. The SLNs were also sonicated using bath ultrasound for 4 min for better homogenization. 

***Characterization of IVM-SLNs***

The polydispersity index (PDI) and size were determined using the Dynamic Light Scattering (DLS) method. The zeta potential of nanoparticles was also measured with a Nanosizer (Malvern, UK). A Shimadzu IR2000 (Japan) with a scan rate of 8/cm for 100 scans was used for scanning the Fourier transform infrared spectroscopy (FTIR) in the 500–4000 cm^-1^ range. Using the Differential scanning calorimetry (DSC) instrument SDT Q 600 (V20.9 Build 20), DSC thermograms were carried out at 10 to 200 °C with a temperature range of 10 °C/min. The IVM-SLNs XRD pattern was scanned utilizing an X-ray diffractometer (PW1730, 40 kV, 30 mA, Philips, Netherlands) with a step size of 0.05° and a time per step of 1 sec.

***Determination of drug entrapment efficiency and drug loading***

Using a UV-vis spectrometer, drug loading (DL) and drug encapsulation efficiency (DEE) were determined in IVM-SLNs. Also, evaluation of IVM concentration in the nanoparticles was performed through IVM calibration curve at 245 nm, and using UV absorption of several standard IVM ethanolic solutions. After centrifugation of freshly prepared NPs (40,000 rpm for 30 min), supernatants were removed to determine free IVM in the suspensions.

***Calculations of DL and DEE were performed utilizing the presented formulas:***

DL (%) $=\frac{\mathrm{Total IVM}-\mathrm{free IVM}}{\mathrm{IVM}-\mathrm{SLN NPs weieght}}=\frac{\mathrm{Total IVM}-\mathrm{free IVM}}{\mathrm{IVM}-\mathrm{SLN NPs weieght}}$× 100

DEE (%) $=\frac{\mathrm{Total IVM}-\mathrm{free IVM}}{\mathrm{Total IVM}}=\frac{\mathrm{Total IVM}-\mathrm{free IVM}}{\mathrm{Total IVM}}$**×** 100

**Drug release of IVM-SLNs**

The release of IVM-SLN nanoparticles was carried out using the dialysis bag diffusion method. In the dialysis bag (cut off 12 kDa), IVM-SLNs were inserted. The dialysis bag was placed in 50 ml PBS (pH 7.4) and was kept at room temperature with constant stirring (500 rpm). At different time intervals during 48 hr, 2 ml was removed and 2 ml fresh was added to the system. Then by spectrophotometric analysis, the absorbance of the samples was monitored at 245 nm and the release at different time intervals was calculated.

***Animals***

Twenty-eight male C57BL/6 mice (8 weeks old) weighing 25±2 g were purchased from the Pasteur Institute of Iran. Mice were kept under laboratory conditions at 25 °C, a 12-hr light/dark cycle, and were fed standard chow *ad libitum* according to the standard instructions for working with animals of Kermanshah University of Medical Sciences, Iran (ethical number: IR.KUMS.AEC.1401.032).

***Induction of cuprizone-demyelination***

The addition of CPZ 0.2% (w/w) to the standard chow of mice was used to induce demyelination. Mice received CPZ-containing food for 6 weeks. Animals were randomly divided into 4 groups of 7 specimens each: 1) Control group: received a standard diet, was used for 6 weeks; 2) CPZ group: received a diet supplemented with 0.2% (w/w) CPZ for 6 weeks; 3) IVM group: received a diet supplemented with 0.2% (w/w) CPZ for 6 weeks and IVM (1 mg/kg/day) by intraperitoneal (IP) injection during the weeks 5 and 6 of the experiment; 4) Nano-IVM (1 mg/kg/day, IP) administered during the weeks 5 and 6 of the experiment (36). The weight of mice was measured each week during the experiment. 

***Behavioral tests***

*Motor tests*

To evaluate the motor balance and coordination of mice, open-field, pole, and rotarod tests were carried out at the end of the study according to the previous studies (37, 38). All three tests were repeated three times at an interval of ten minutes. 

*Analgesic tests*

The elevation of the neuronal response threshold to non-painful stimuli is known as allodynia and is characteristic of MS (39). Hot plate and acetone tests were used to evaluate the hot and cold thermal allodynia, respectively, according to the previous studies (40, 41). 

***Tissue preparation***

On the last day of the experiment and after the behavioral tests, animals were anesthetized and euthanized under deep anesthesia with IP injections of ketamine (60 mg/kg) and xylazine (10 mg/kg). The brains were fixed in 10% formaldehyde and used for histological assays. Hematoxylin and eosin (H&E) staining of the prefrontal cortex (PFC), immunohistochemistry (IHC) of the corpus callosum for TRPA1, p-NF-kB p65, GFAP, and MBP antibodies, and luxol fast blue (LFB) stain of the corpus callosum were carried out.

***Immunohistochemistry (IHC) assay***

IHC was done to analyze the corpus callosum levels of TRPA1, NF-kB p65, GFAP, and MBP. Five µm paraffin-fixed slides of the corpus callosum were deparaffinized with xylene, processed through downgraded alcohols, and rehydrated. Then, antigen retrieval was performed with citrate buffer (pH = 7.4). After washing with TBS plus 0.03% Triton X-100, the slides were blocked in 10% normal serum for 2 hr at room temperature and incubated with primary antibodies (dilutions 1:100) overnight at 4 °C. Next, the slides were washed 3 times with TBS plus 0.03% Triton X-100 and incubated with goat biotinylated polyvalent secondary antibody (dilution 1:100) for 10 min at room temperature. Then, slides were washed with PBS, and DAB substrate (diaminobenzidine solution) was added. Finally, a counterstain with hematoxylin-eosin was performed. 

***Hematoxylin and eosin (H*** ***&*** ***E) assay***

Five µm slides containing paraffin sections from PFC were deparaffinized and rehydrated with graded ethanol and water. Then, hematoxylin solution was added (for 6 hr, 60 °C) and rinsed with deionized water. After that, eosin staining and dehydration with graded ethanol were carried out. Finally, the samples were dried overnight in a laminar flow hood.

***Luxol fast blue (LFB) staining***

The degree of demyelination in the CNS was determined using the LFB staining. The density of the blue color is related to the healthy myelin of neurons (42). Paraffin-embedded corpus callosum sections were deparaffinized with ethanol 95% and stained with 0.1% LFB (overnight, 56 °C). Then, the slides were washed with 70% ethanol and distilled water. Differential staining was done with 0.05% lithium carbonate and 70% ethyl alcohol. The slides were cleared with xylene and evaluated by a light microscope. The amount of demyelination was estimated using the ImageJ software.

***Statistics assay***

GraphPad Prism 9 (San Diego, CA, USA) was used for data analysis. One-way analysis of the variance (ANOVA) and two-way ANOVA (for weight changes during 6 weeks) followed by Tukey’s *post hoc* test were used. Data were shown as mean ± SEM and *P<*0.05 was reflected as statistically significant. The quantification of the histological assays was done by ImageJ software for Windows. 

## Results

***Dynamic light scattering (DLS) analysis***

The mean size of IVM-SLNs (nano-IVM) in colloidal solution was 573 nm with a PDI of 0.32. Measurement of zeta potential is significant to understand the morphological status of the nanoparticle surface and predict the long-term kinetic stability of the formulation (43). This factor shows the degree of repulsion among particles with the same charge in dispersions. It is also responsible for colloidal scattering dispersions (43, 44). Analysis of zeta potential showed that nanoparticles are stable with a negative zeta potential value of -31 mV. The negative value may indicate the existence of fatty acids in the preparation of SLNs, which affords fixed hydrophobic interactions (44). 

***Fourier transform infrared spectroscopy (FTIR) analysis***

**Figure 1:**
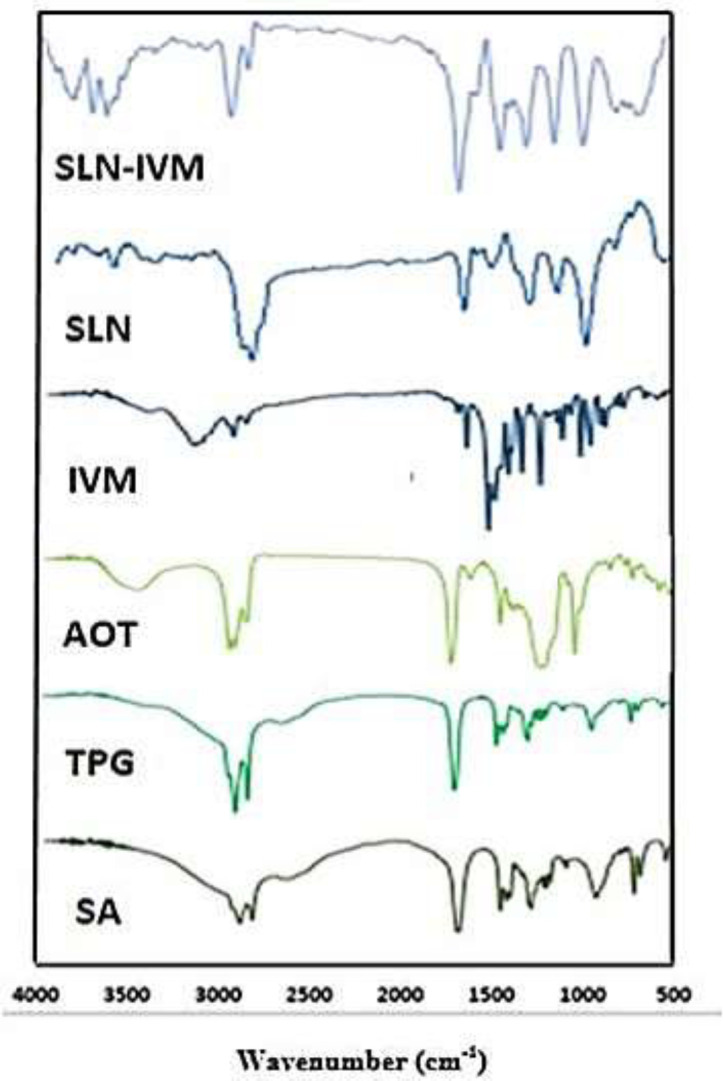
FT-IR spectra of SA, TPG, AOT, IVM, SLN, and IVM-SLNs (nano-IVM)

FTIR analysis was used to confirm IVM loading in SLNs. Figure 1 shows the FTIR for pure IVM, SLN, and IVM-SLNs. Several peaks of lipids are observed in the SLNs structure at wavelengths of 1290, 1463, and 1703-1730 cm^-1^ depending on the C = O tensile bands, indicating that SLN is synthesized (35). In the case of pure IVM, the wavenumber at 3483 cm^-1^ indicates OH tension, and a sharp absorption band appearing at about 2935 cm^-1^ corresponds to CH tension, whereas 1732–1703 cm^-1^ corresponds to OH tension. Power can be attributed to the tensile bond of the C6 ring (45). Comparing the range of SLN systems with IVM-SLNs, it was found that drug entry into the system caused minor changes in the peaks of the individual IVM-SLNs compared to IVM, indicating that the drug had entered SLN. Specifically, the peaks in the SLN are displaced by a slight change in the order of the IVM-SLNs points. Therefore, since no new peak has been created and the peak has not disappeared, no chemical interactions between the system and the drug, and the drug and the system have retained their nature. Therefore, these results confirm the complete confinement of IVM in SLNs. 

***Differential scanning calorimetry (DSC) analysis***

**Figure 2:**
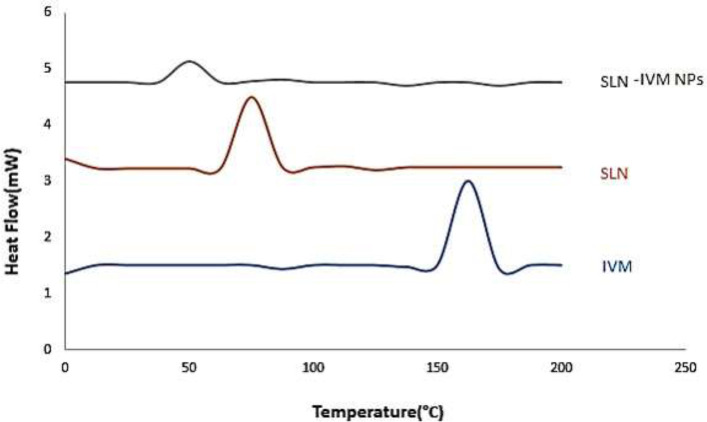
DSC thermograms of IVM, SLN, and IVM-SLNs (nano-IVM)

DSC is a standard procedure that gives us accurate data about any changes in the enthalpy of the system from every exothermic or endothermic phenomenon that occur in the system, as well as investigating the melting and recrystallization behavior of crystalline material such as the SLNs (35, 46). In this thermal decomposition method, alterations in the heat capacity of a material as a function of temperature are continuously measured. The thermogram of pure IVM at 162.5 °C shows an endothermic peak that is its melting point (Figure 2). The SLN thermocouple peak is also shown at 75 °C. In our study, the characteristic endothermic peak at 162.5 °C, due to the melting of IVM, disappears in the DSC thermogram of IVM-SLNs, demonstrating that the drug is loaded in the nanoparticles. 

***X-ray diffraction (XRD) analysis***

**Figure 3:**
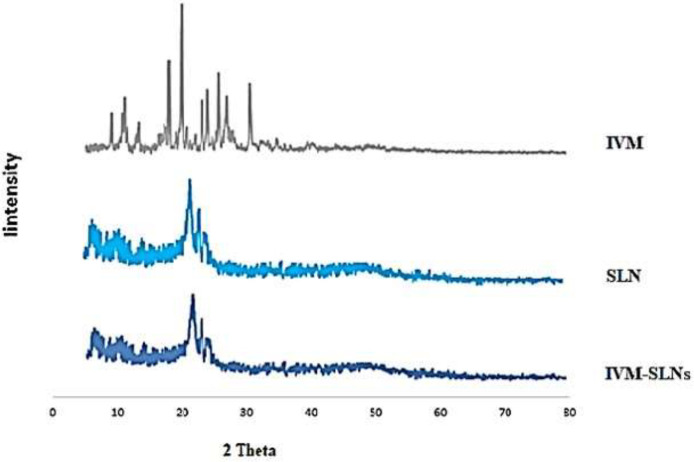
X-ray patterns of IVM, SLN, and IVM-SLNs (nano-IVM)

The diffraction pattern of pure IVM, SLN, and IVM-loaded SLNs was analyzed (Figure 3). The diffraction patterns of SLN and IVM-loaded SLNs showed that less arranged crystals are predominant and, therefore, the amorphous state helps the higher capacity of drug loading. The sharp peaks of IVM (17.97, 19.87, 25.67, and 30.62^0^), which indicate its crystalline nature, are not existent in the diffraction pattern of IVM-loaded SLNs, showing drug loading in SLNs as well as in molecular or amorphous dispersion. Also, there is not much difference in the diffraction patterns of SLNs and IVM-loaded SLNs, revealing that IVM loading did not alter the nature of SLNs (47). 

***Drug entrapment efficiency (DEE) and drug loading (DL)***

The amount of IVM present in the supernatant was determined using a calibration curve at 245 nm. Then, according to the IVM calibration curve, weight values of IVM-loaded SLN NPs and equations DL and DEE were calculated. The results revealed that DEE and DL were 87% and 20%, respectively.

***In vitro drug release studies***

**Figure 4:**
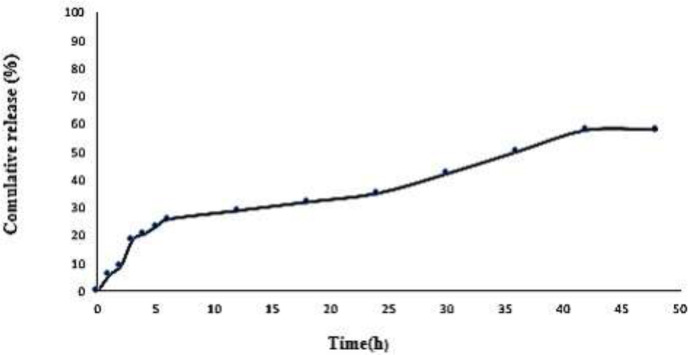
Release percentage of IVM from IVM-SLNs (nano-IVM)

In this study, drug release from SLN NPs with a size of 570 nm is considered. The release profile of IVM-loaded SLN in PBS at pH 7.4 is shown in Figure 4. The results show that drug release after 48 hr, at pH 7.4 is close to 58%.

***Animal study***

*Bodyweight changes*

**Figure 5:**
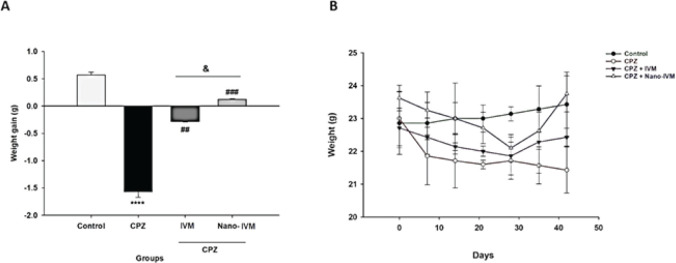
Changes in body weight (n=7)

As shown in Figure 5A-B CPZ exposure resulted in a significant weight loss after the first week maintained during the next five weeks compared to the control group (*P*<0.0001). However, IVM and nano-IVM treatment reversed the weight loss induced by CPZ (*P<*0.01 and *P<*0.001, respectively). The nano-IVM group had no weight loss and increased body weight compared to the IVM group (*P<*0.05). 

*Behavioral tests*

Pole test 

**Figure 6:**
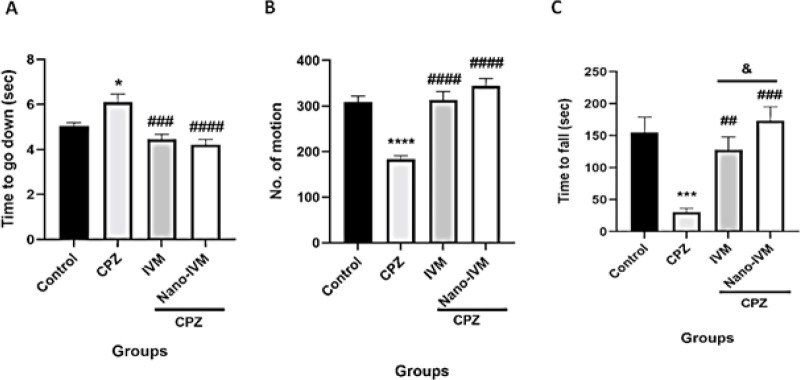
Locomotor activity tests (n=7)

The results of the pole test to evaluate the motor balance after exposure to CPZ and the protective effects of IVM and nano-IVM treatments are presented in Figure 6A. The CPZ group showed markedly lower performance compared to the control group taking longer to reach the floor of the apparatus (*P<*0.05). Treatment with IVM and nano-IVM significantly improved the performance compared to the CPZ group (*P<*0.001 and *P*<0.0001, respectively). 

Open field test 

CPZ exposure significantly reduced the number of movements performed by the mice and increased the time of immobility (*P<*0.0001, Figure 6B). Both IVM and nano-IVM treatments increased the number of movements and reduced the immobility induced by CPZ (*P<*0.0001).

Rotarod test 

The alteration in motor balance was also evaluated by the rotarod test (Figure 6C). Mice of the CPZ group spent a shorter time on the rotarod apparatus than the control group (*P<*0.001). The mice that received IVM and nano-IVM treatments improved motor coordination compared to the CPZ group and spent longer time on the apparatus (*P<*0.01 and *P<*0.001, respectively). The treatment with nano-IVM resulted in better motor coordination when compared to the IVM group (*P<*0.05). 

Hot plate test 

**Figure 7:**
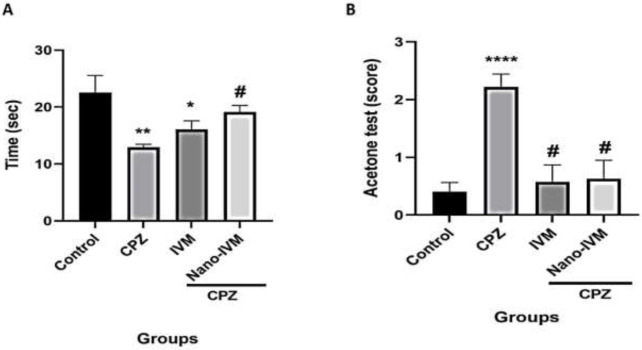
Analgesic tests (n=7)

The results of the hot plate test done at the end of the experiment were presented in Figure 7A. A significant reduction in the latency time for licking the hind paws, shaking, or jumping was observed in the CPZ and IVM groups (*P<*0.01 and *P<*0.05, respectively). The treatment with nano-IVM increased significantly the time threshold on the hot plate and reduced the thermal allodynia compared to the CPZ group (*P<*0.05).

Acetone test

The evaluation of cold allodynia with the acetone test done at the end of the experiment was presented in Figure 7B. The cold reaction score was significantly higher in the CPZ group than in the control (*P<*0.0001). The treatment with IVM and nano-IVM markedly reduced the cold reaction score in the acetone test compared to the CPZ group (*P<*0.05).

*Luxol fast blue staining*

**Figure 8:**
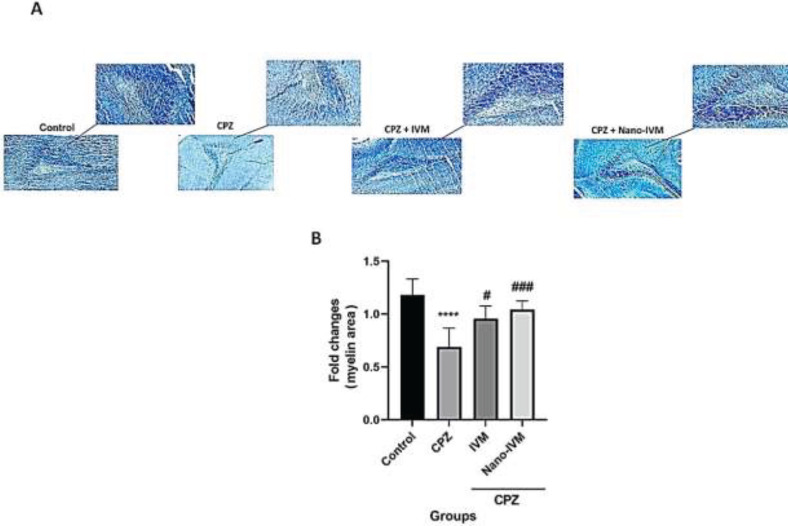
LFB staining of the corpus callosum (x 40 and x 100) (n = 4)

Myelin staining to evaluate the demyelination in the carpus callosum was carried out after exposure to CPZ, IVM, and nano-IVM treatments (Figure 8A-B). The CPZ intoxication resulted in a much lower blue color intensity in the neurons of the carpus callosum than that in the control group (*P<*0.0001), demonstrating the myelin degeneration effect of CPZ. Treatment with IVM and nano-IVM significantly reduced the myelin degeneration induced by cuprizone and increased significantly the blue color intensity compared to the CPZ group (*P<*0.05 and *P<*0.001, respectively).

*Hematoxylin and eosin assay*

**Figure 9:**
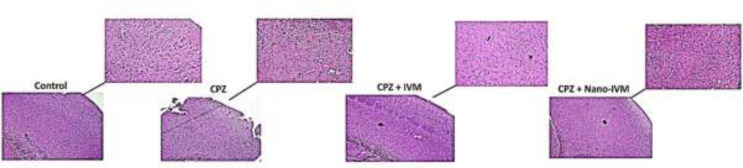
Hematoxylin and eosin staining of the prefrontal cortex (x 40 and x 100) (n = 4)

H&E staining of the PFC of the control group (Figure 9) showed a normal structure of the cortex layers. The pia mater (PM) that covers the cortex was thin and regular, and the molecular layer (ML) had a normal structure, evidencing a regular arrangement. The inner layers contained pyramidal cells (PC) with vesicular nuclei and basophilic cytoplasm. Also, normal neuropil-containing capillaries and some microglia were detected. Conversely, stained PFC of the CPZ group showed the thickened PM and ML layer, and the arrangement of cellular layers was disrupted. In this CPZ group, hypercellularity was also observed in the cortex. Some of the PC were irregular in shape, and vacuoles indicated apoptosis was formed around them. Moreover, the presence of vacuolated neuropils was found. PFC staining of the IVM and nano-IVM treated groups showed a significant improvement in the histological structure of PFC. The thickness of PM was normalized, and the thickness of ML was reduced when compared to the CPZ group. 

*Immunohistochemistry assay of TRPA1, NF-kB p65, GFAP, and MBP*

**Figure 10:**
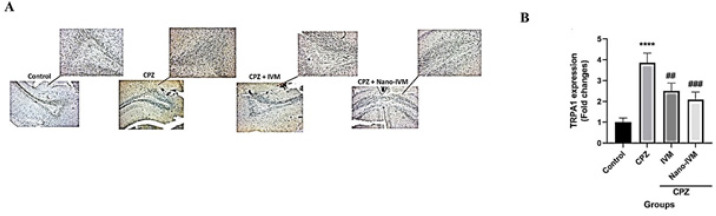
A: TRPA1 immunohistochemistry staining (IHC) of the corpus callosum (x 40 and x 100) (n =4). B: IHC staining optical intensity data by ImageJ

**Figure 11:**
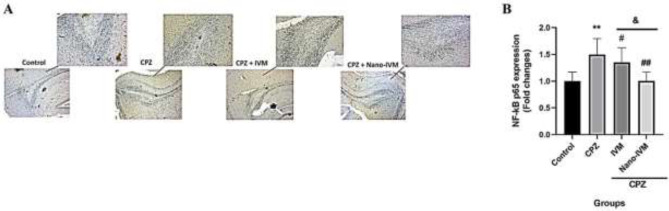
A: NF-kB p65 immunohistochemistry staining (IHC) of the corpus callosum (x 40 and x 100) (n = 4). B: IHC staining optical intensity data by ImageJ

**Figure 12:**
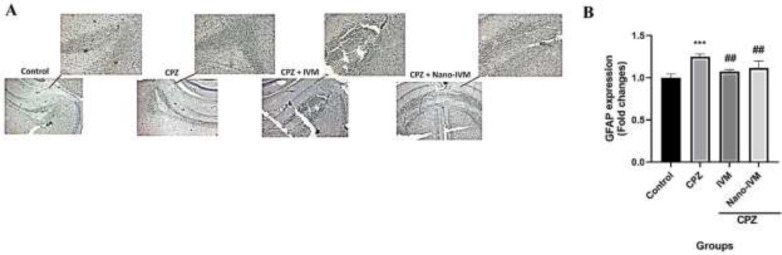
A: GFAP immunohistochemistry staining (IHC) of the corpus callosum (x 40 and x 100) (n = 4). B: IHC staining optical intensity data by ImageJ

**Figure 13:**
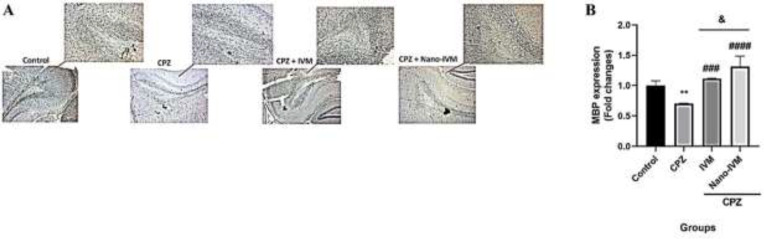
A: MBP immunohistochemistry staining (IHC) of the corpus callosum (x 40 and x 100) (n = 4). B: IHC staining optical intensity data by ImageJ

To observe the effect of CPZ intoxication, IVM and nano-IVM treatments on corpus callosum expression of TRPA1, NF-kB p65, GFAP, and MBP, immunohistochemical staining (IHC) were performed (Figures 10–13, respectively). In the CPZ group, the levels of TRPA1, GFAP, and NF-kB expression were significantly higher than that in the control group (*P<*0.0001, *P<*0.001, and *P<*0.01, respectively). In contrast, the corpus callosum levels of MBP in the CPZ group were markedly reduced with respect to the control group (*P<*0.01), indicating damage of myelin induced by CPZ. The treatment with IVM and nano-IVM considerably protected from myelin injury or stimulated myelin regeneration via reducing the corpus callosum levels of TRPA1, NF-kB p65, and GFAP compared to the CPZ group (*P<*0.01, *P<*0.05, and *P<*0.01, respectively for IVM group and *P<*0.001, *P<*0.01, and *P<*0.01 for nano-IVM group). On the contrary, in the IVM and nano-IVM groups, the expression levels of MBP were significantly higher than that in the CPZ group (*P<*0.001 and *P<*0.0001, respectively). The modulations of the levels of NF-kB p65 and MBP were significantly stronger in the nano-IVM group than that observed in the IVM group (*P<*0.05). 

## Discussion

The main goal of this research was to assess the neuroprotective effects of IVM and nano-IVM in CPZ-induced demyelination mice and to observe the role of the TRPA1/NF-kB/GFAP signaling pathway. One of the most common animal models of demyelination representing MS pathology is the CPZ model which induces motor incoordination, myelin degeneration, and activation of glial cells in the CNS (48). 

CPZ exposure for 6 weeks resulted in motor impairment and behavioral alterations evidenced by various tests, including pole, rotarod, open field, hot plate, and acetone tests as was reported in previous studies (49, 50). Behavioral changes induced by CPZ, such as motor abnormalities, imbalance coordination as well as body weight loss were related to myelin degeneration (51). In the current study, CPZ feeding caused significant body weight loss, which was ameliorated by IVM and entirely recovered by nano-IVM treatment. In addition, IVM and nano-IVM administration improved behavioral alterations and motor balance in all performed tests demonstrating a reduction in the adverse effects of CPZ on myelin. Histological analysis (H&E and LFB staining) evidenced that IVM and nano-IVM normalized the morphological alteration induced by CPZ. Specifically, the nano-formulation of IVM improved its neuroprotective effects via reducing demyelination or maybe inducing myelin regeneration. 

In a study that aimed to discover a neuroprotective drug for ischemic stroke, it was shown that IVM has neuroprotective effects (52). It has been reported that IVM at high concentrations (µM range), can operate as an allosteric moderator of several channels such as receptors of γ-aminobutyric acid A (GABAA) from humans, rodents, and chickens; receptors of human purinergic (P2X420 and P2X7), as well as receptor of human glycine (53-55). In 2018, a study by Cairns and colleagues was conducted to investigate the role of IVM in mammalian nerve repair. The results showed that IVM through inducing fibroblasts results in the promotion of peripheral nerve regeneration (55). In another study, the role of IVM in the hyperinnervation of primary eye tissue in Xenopus laevis frogs has been shown (56). Moreover, findings of an *in vivo* study showed that IVM can limit the excitotoxicity caused by the AMPA receptor to 10% in the development of amyotrophic lateral sclerosis (ALS) in a transgenic mouse model and thereby increase its life (57). 

MBP is one of the most important proteins involved in the myelination process of nerves in the CNS. MBP, which is located on the myelin sheath, preserves the correct structure and function of myelin (58). Several studies have indicated a correlation between MBP antibodies and MS pathology (59, 60). In this research, CPZ intoxication markedly resulted in a decreased level of MBP in the corpus callosum that was reversed by IVM and nano-IVM with a better effect of nano-IVM, indicating the preventive role of IVM in reducing demyelination.

NF-kB, a transcription factor, controls the production of cytokines and cell survival (61). NF-kB was activated by stimuli such as cytokines, free radicals, oxidative stress, and microbial antigens (62). NF-κB subunits include p50, p65, and IkB (63). Numerous factors such as ubiquitination, phosphorylation, and degradation of the inhibitor of NF-κB (IκBα), lead to nuclear translocation of p50- p65 subunits of NF-κB, followed by p65 phosphorylation, methylation, acetylation, gene transcription, and DNA binding. So, agents that can restrain protein kinases, protein phosphatases, proteasomes, ubiquitination, acetylation, methylation, and DNA binding steps have been identified as NF-κB inhibitors (64). It has been shown that improper regulation of NF-κB is associated with autoimmune diseases and inflammation (63). Up-regulation of NF-kB induces the expression of pro-inflammatory cytokines as well as inducible nitric oxide synthase (iNOS). In the present work, the levels of NF-kB p65 were significantly greater in the CPZ group. These data agree with previous studies reporting the activation of NF-kB by CPZ (49, 65). Nevertheless, IVM and nano-IVM treatment (with the significantly better effect of nano-IVM) reduced the expression of NF-kB. In line with these data, several *in vitro* and *in vivo* studies have shown that IVM reduced the levels of TNF-α, IL-2, and IL-6 via suppressing nuclear NF-kB translocation (66, 67). 

TRPA1 is another essential target involved in MS and neuropathic pain in MS (68). TRPA1, a membrane cation channel located in PNS and CNS astrocytes, is activated by changes in temperature, free radicals, and itch, and its activation causes pain. It has been indicated that TRPA1 knockout mice showed reduced demyelination induced by CPZ intoxication (69, 70). Several *in vivo* and *ex vivo* studies have demonstrated the link between TRPA1 with neuroinflammation caused by beta-amyloid in the Alzheimer model and demyelination induced by ischemia (71, 72). A study on bronchial epithelial cells has shown that the activation of TRPA1 by LPS activates ROS generation, In addition, the NF-kB, translocation to the nucleus was inhibited by TRPA1 antagonist (HC030031) as well as in trpa1^–/–^ Mice (73). Neurotoxicity effects of TRPA1 activation, such as increased levels of caspase-3, TNF-α, and IL-1β, were related to the MAPK/NF-kB signaling pathway and were blocked by TRPA1 antagonist HC-030031, NF-kB inhibitor (BAY 11-7082), and MAPK inhibitor (U0126) (74). Reciprocally, in an *in vitro* study, it was illustrated that the pro-inflammatory cytokine TNF-α increased the expression of TRPA1 via MAPK/NF-Kb signaling pathway activation (75). In the current research, CPZ-feeding mice revealed a significant elevation of corpus callosum TRPA1 expression that was positively related to NF-kB p65 expression. Treatment with IVM and nano-IVM markedly reduced the expression of TRPA1 induced by CPZ. Samways *et al*. have shown that IVM reduced the levels of calcium in mouse cerebellar microglia via inhibition of ligand-gated ion channels such as P2X4 and TRPA1 (76).

One of the main markers of astrogliosis occurring in MS plaque is a glial fibrillary acidic protein (GFAP) which is expressed in the CNS, PNS, and ENS. GFAP level increases in inflammatory conditions such as neurological damage and inflammatory bowel disease (IBD) (77). GFAP serum and CSF levels are directly linked to MS severity and progression (78). It has been indicated that inflammatory diseases of CNS such as neuromyelitis optica and MS showed high levels of GFAP in CSF (79). Glial scar occurring in neurodegenerative diseases is formed by the interaction between astrocytes and fibrous tissue around the central damage core and is partly triggered by GFAP elevation (80). IHC of the spinal cord and brain sections have demonstrated that elevation of GFAP and activation of astrocytes was observed several days before, leading to clinical symptoms (81). In this research, the level of GFAP in the corpus callosum was markedly higher in the CPZ group contributing to the inflammation and astrogliosis. However, IVM and nano-IVM reduced the GFAP expression and improved neurological dysfunction. 

SA: Stearic acid, TPG: Glycerol tripalmitate, AOT: Dioctyl sulfosuccinate sodium salt, IVM: Ivermectin, SLN: Solid lipid nanoparticle

DSC: Differential scanning calorimetry, IVM: Ivermectin, SLN: Solid lipid nanoparticle

IVM: Ivermectin, SLN: Solid lipid nanoparticle

IVM: Ivermectin, SLN: Solid lipid nanoparticle

A: weight gain at the end of experiment, B: body weight alteration during the experiment

CPZ: Cuprizone, IVM: Ivermectin, Nano-IVM: Nano-ivermectin. ****P<0.0001 vs the control group, ##*P*<0.01 and ###*P*<0.001 vs the CPZ group

The data were shown as mean ± SEM.

A: Pole test, indicates time to fall away from the pole; B: Open field test, indicates the number of motions during 5 min; C: Rotarod test, indicates time to fall

CPZ: Cuprizone, IVM: Ivermectin, Nano-IVM: nano-ivermectin

*P<0.05, ***P<0.001, and ****P<0.0001 vs the control group; ##*P*<0.01, ###*P*<0.001, and ####*P*<0.0001 vs the CPZ group

The data were shown as mean ± SEM.

A: Hot plate test; B: Acetone test

CPZ: Cuprizone, IVM: Ivermectin, Nano-IVM: Nano-ivermectin

*P<0.05, *P<0.01, ***P<0.001, and ****P<0.0001 vs the control group; #*P*<0.05 vs the CPZ group

The data were shown as mean ± SEM.

A: Cuprizone decreased the intensity of the blue color meaning demyelination that was improved with IVM and nano-IVM treatment. B: LFB staining optical intensity data by ImageJ LFB: Luxol fast blue, CPZ: Cuprizone, IVM: Ivermectin, Nano-IVM: Nano-ivermectin

****P<0.0001 vs the control group, #*P*<0.05 and ###*P*<0.001 vs the CPZ group

The data were shown as mean ± SEM.

CPZ: cuprizone, IVM: ivermectin, Nano-IVM: nano-ivermectin

TRPA1: Transient receptor potential A, CPZ: Cuprizone, IVM: Ivermectin, Nano-IVM: Nano-ivermectin

****P<0.001 vs the control group; ##*P*<0.01 and ###*P*<0.001 vs the CPZ group

The data were shown as mean ± SEM.

**P<0.01 vs the control group; #*P*<0.05 and ##*P*<0.01 vs the CPZ group

The data were shown as mean ± SEM.

CPZ: Cuprizone, IVM: Ivermectin, Nano-IVM: Nano-ivermectin

***P<0.001 vs the control group; ##*P*<0.01 vs the CPZ group

The data were shown as mean ± SEM.

CPZ: Cuprizone, IVM: Ivermectin, Nano-IVM: Nano-ivermectin

**P<0.01 vs the control group; ###*P*<0.001 and ####*P*<0.0001 vs the CPZ group

The data were shown as mean ± SEM.

## Conclusion

The present results establish for the first time that IVM has a neuroprotective effect on CPZ-induced demyelinated C57BL/6 mice through reducing behavioral dysfunctions and neuroinflammation, and induction demyelination. In addition, the results reveal the role of the TRPA1/NF-kB/GFAP signaling pathway in the neuroprotective effect of IVM. These data show that both IVM and its nano-formulation can be candidates for further clinical studies on demyelinating disorders and improve MS symptoms.

## Authors’ Contributions

S S helped conceive the study, provided methodology and supervision, and contributed to writing the original draft, review, and editing. SD A, SZ H, and S K performed data curation and helped write the original draft. A S helped validate, investigate, write, review, and edit. TN performed visualization and investigation, and contributed to writing, reviewing, and editing. A D and J E provided software analysis, validation, and writing, reviewing, and editing. 

## Approval by the Medical Ethical Committee

The study was done according to the standard instructions for working with animals of Kermanshah University of Medical Sciences, Iran (ethical number: IR.KUMS.AEC.1401.032).

## Data Availability

Data will be made available upon request.

## Conflicts of Interest

None. 
